# Generation of an arrayed CRISPR-Cas9 library targeting epigenetic regulators: from high-content screens to *in vivo* assays

**DOI:** 10.1080/15592294.2017.1395121

**Published:** 2018-01-12

**Authors:** Tristan Henser-Brownhill, Josep Monserrat, Paola Scaffidi

**Affiliations:** a Cancer Epigenetics Laboratory, The Francis Crick Institute, 1 Midland Road, London NW1 1AT, UK; b UCL Cancer Institute, University College London, London WC1E 6DD, UK

**Keywords:** epigenetics, CRISPR, arrayed library, sgRNA, high-content, DNA methylation, cancer biology, chromatin, chromatin remodeling, histone modifications

## Abstract

The CRISPR-Cas9 system has revolutionized genome engineering, allowing precise modification of DNA in various organisms. The most popular method for conducting CRISPR-based functional screens involves the use of pooled lentiviral libraries in selection screens coupled with next-generation sequencing. Screens employing genome-scale pooled small guide RNA (sgRNA) libraries are demanding, particularly when complex assays are used. Furthermore, pooled libraries are not suitable for microscopy-based high-content screens or for systematic interrogation of protein function. To overcome these limitations and exploit CRISPR-based technologies to comprehensively investigate epigenetic mechanisms, we have generated a focused sgRNA library targeting 450 epigenetic regulators with multiple sgRNAs in human cells. The lentiviral library is available both in an arrayed and pooled format and allows temporally-controlled induction of gene knock-out. Characterization of the library showed high editing activity of most sgRNAs and efficient knock-out at the protein level in polyclonal populations. The sgRNA library can be used for both selection and high-content screens, as well as for targeted investigation of selected proteins without requiring isolation of knock-out clones. Using a variety of functional assays we show that the library is suitable for both *in vitro* and *in vivo* applications, representing a unique resource to study epigenetic mechanisms in physiological and pathological conditions.

## Introduction

Clustered regularly interspaced short palindromic repeats (CRISPR) in combination with CRISPR-associated proteins (Cas) provide a defense mechanism to bacteria and archaea against foreign genetic material [1,2]. Because of its ability to cleave DNA in specific regions, the CRISPR/Cas9 system has recently been adapted to allow precise genome editing in various organisms [3]. Expression of the Cas9 nuclease into a cell, in conjunction with a synthetic small guide RNA (sgRNA), allows targeting of the Cas9/sgRNA complex to a desired genomic location and induction of double-strand breaks (DSBs) that will be repaired by either non-homologous end-joining (NHEJ) or homologous recombination (HR) [3,4]. Depending on the experimental setting, the CRISPR/Cas9 system can be used to induce gene knockout (KO) [5,6], introduce or correct specific mutations in the genome [6–8], delete regulatory regions [9], induce translocations [10], activate or repress gene expression [11], pull-down specific genomic regions [12], or visualize genomic loci [13].

A major application of the CRISPR/Cas9 system is performing loss-of-function screens [3], [14]. NHEJ is the predominant repair pathway in mammalian cells and often results in the formation of insertions or deletions (in/dels) that disrupt gene function [15]. The ability to induce complete loss of the target protein through gene knockout offers several advantages compared to RNA interference-based methods, including improved signal-to-noise ratio and lower off-target effects [16],[17]. The CRISPR/Cas9 system has recently been used in selection screens to identify, among others, essential genes in mammalian cells [5],[18], synthetic lethal genes,[19] HIV host dependency factors [20], genetic vulnerabilities in cancer [21],[22], and regulatory networks in immune cells [23]. These screens utilized pooled sgRNA libraries generated by cloning chip-synthesized oligonucleotides covering the entire human or mouse transcriptome into lentiviral vectors. Upon transduction of the libraries into cells, sgRNAs inducing a selectable phenotype were identified by next-generation sequencing.

Pooled libraries are particularly suitable for positive selection screens, in which KO of important genes confers a strong selective advantage and enrichment of the corresponding sgRNAs. The CRISPR/Cas9 system also enables drop-out screens and has been used to identify dependencies of cancer cells [5],[18]. Due to the high rate of false positives in selection screens, only substantial phenotypic changes induced by gene KO can be detected, and subtler phenotypes are typically missed. Systematic interrogation of all proteins involved in a pathway or a process is therefore not possible. Furthermore, pooled libraries are not suitable when complex or microscopy-based readouts are used, for which libraries containing individual sgRNAs in an arrayed format are required. Although whole-genome arrayed libraries that allow comprehensive interrogation of gene function have been generated [24], they require large amounts of reagents, automated equipment and dedicated facilities. Moreover, many screens do not require examination of the whole genome and focused libraries targeting classes of related proteins may provide a more suitable tool to effectively address specific questions.

Epigenetic mechanisms play a critical role in development, tissue homeostasis and contribute to various diseases, including cancer, syndromes associated with neurological defects and heart disease [25],[26]. Owing to their inherent reversibility, epigenetic mechanisms represent promising therapeutic targets and identification of novel epigenetic regulators involved in disease may open new avenues for therapeutic intervention. Here, we report the generation and characterization of an sgRNA library targeting most known human epigenetic regulators with multiple guides, suitable for both selection and high-content screens, and compatible with a variety of *in vitro* and *in vivo* assays.

## Results

### Library design

**Figure 1.**
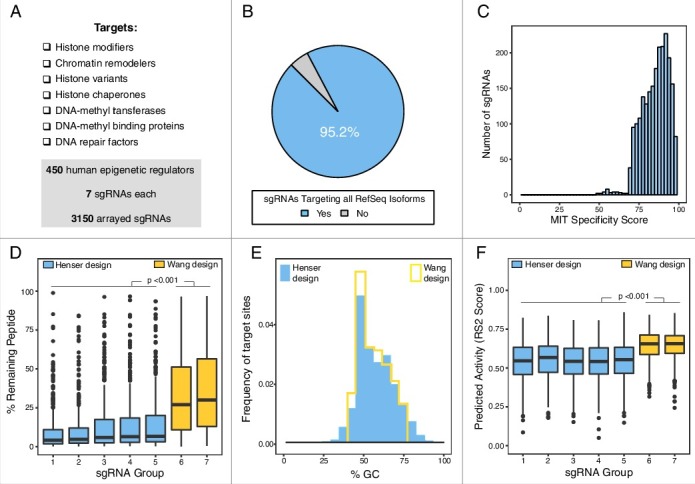
**sgRNA library design** **A**. Basic information about the genes targeted in the sgRNA library. The number and the classes of targeted genes are indicated. **B**. Relative abundance of designed sgRNAs (groups 1–5) targeting all RefSeq isoforms of the corresponding genes (blue). **C**. Distribution of MIT specificity score across the designed sgRNA groups 1–5. **D**. Distribution of targeted sites within genes, comparing newly designed sgRNAs (Henser design, blue) and sgRNA selected from the library designed by Wang et al. [18] (Wang design, yellow). Groups 1–5 sgRNAs (Henser design) target the most 5’ exons, resulting in the formation of short truncated proteins. In/dels induced by groups 6–7 sgRNAs (Wang design) lead to longer truncated proteins, possibly retaining partial functionality. *P* value: 2-way ANOVA. **E**. Distribution of GC content of the target sequences across the indicated sgRNA groups. **F**. Predicted activity of the indicated sgRNA groups based on the Azimuth algorithm. *P* value: 2-way ANOVA.

Epigenetic regulators to be targeted were selected based on Gene Ontology, including proteins involved in chromosome organization, chromatin modification, and regulation of DNA methylation. Additional proteins were included based on overall similarity with known epigenetic regulators or the presence of protein domains predicted to bind to or modify chromatin (e.g., chromodomain, bromodomain or SET domain). Selected proteins involved in DNA damage response and repair were also included in the list of targets. All together, 450 genes were selected for targeting (Fig. 1A, Supplementary Table 1).

For each gene, we designed 5 different sgRNAs (sgRNA groups 1–5) using the CRISPR MIT Designer (crispr.mit.edu) [27]. Where possible, sgRNAs were designed to target the first translated exon common to all RefSeq isoforms of each gene (Fig. 1B and Supplementary Fig. 1A). To minimize off-target effects, we preferentially selected sgRNAs with scores of at least 70, a value that typically characterizes highly specific sgRNAs [27] (Fig. 1C). When targeting of a shared exon with a specific sgRNA was not possible, the most 5’ exon of the primary RefSeq variant was used. (Supplementary Fig. 1A). As an additional criterion, whenever possible, we ensured that targets had at least 3 non-overlapping guides. Based on the above strategy, the designed set of guides comprised 98% of sgRNAs with score ≥70 (Fig. 1C), 74% of sgRNAs targeting the N-terminus part of the corresponding proteins (% of remaining peptide ≤25) (Fig. 1D, blue boxes), and 90% of sgRNAs with optimal GC content (35-70%) [28] (Fig. 1E, blue histogram). Furthermore, analysis of the designed sgRNAs using an algorithm that predicts guide efficacy [29] showed that 82% of the sgRNAs had an Azimuth RS2 score ≥0.4 (range: 0.04-0.81), indicating high predicted activity (Fig. 1F, blue boxes). Similar results were obtained analyzing the sgRNA sequences using RNA Scorer 2.0, a different predictive algorithm[30] (Supplementary Fig. 2A,B). Thus, the designed sgRNA groups 1–5 are predicted to generate short truncated proteins, with minimal off-target effects and high efficacy.

**Figure 2.**
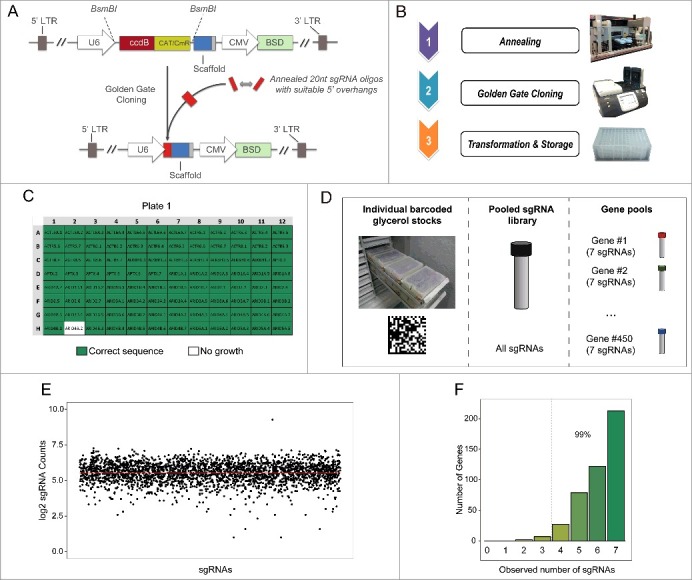
**sgRNA library generation A**. Schematic representation of the vector used to generate the sgRNA library. LTR: Long Terminal Repeat; U6: U6 promoter; ccdB: suicide gene; CAT/CmR: chloramphenicol resistance; BsmBI: restriction enzyme. CMV: cytomegalovirus promoter; BSD: blasticidin resistance. The lower side of the graphics represents the final construct containing the sgRNA (red rectangle). **B**. Summary of the three-step large-scale cloning protocol. **C**. Heatmap showing the fraction of correct constructs in plate 1 of the library as assessed by Sanger sequencing. Only one sgRNA, indicated in white, was not successfully cloned. **D**. Summary of the three different formats in which the library was generated and stored. **E**. Relative abundance of individual sgRNAs as assessed by next-generation sequencing of the pooled library. Each dot represents an individual sgRNA. The red line indicates the median value of sgRNA counts. **F**. Number of genes with the indicated number of designed sgRNAs represented in the library. The percentage of genes targeted by at least 4 sgRNAs is indicated.

For each gene in the library, 2 additional guides (sgRNA groups 6–7) were selected from the whole-genome CRISPR library constructed by Wang et al., [18] which comprises sgRNAs primarily designed based on predicted efficacy. Unlike sgRNAs groups 1–5, the sgRNA groups 6–7 do not target preferentially 5’ exons and showed a more uniform distribution along genes (Fig. 1D, yellow boxes), but had comparable GC content and higher Azimuth RS2 score (Fig. 1E-F). The combined sgRNA groups resulted in a library of 3150 unique sgRNA sequences with 7 sgRNAs per target gene (Supplementary Table 2).

To achieve efficient in/del generation, we chose a selectable lentiviral vector for delivery of sgRNAs into cells. pLenti_BSD_sgRNA, generated by substituting the EGFP coding region of the pLenti plasmid [31] with a cDNA (BSD) encoding resistance to the antibiotic blasticidin, allows efficient expression of sgRNAs under a human U6 promoter (Fig. 2A). To be amenable for large scale cloning, the vector of choice should allow fast and efficient cloning of sgRNAs, and eliminate any background by empty vector. pLenti_BSD_sgRNA contains two *BsmBI* restriction sites, which allow efficient plasmid digestion and insert ligation in a single reaction by Golden Gate cloning. Moreover, the vector contains a *ccdB* suicide gene, which is replaced by the sgRNA upon cloning (Fig. 2A), preventing propagation of the empty vector and allowing highly efficient selection of bacteria transformed by the correct plasmid without needing to plate them on solid medium.

### Library production

For each sgRNA, individual forward and reverse oligos tagged with appropriate 5’ overhangs compatible with the *BsmBI*-based ligation were synthesized in 96-well format (2 × 33 plates). Annealed oligos were cloned into pLenti_BSD_sgRNA by Golden Gate cloning (see methods) (Fig. 2B and Supplementary Fig. 1B). *Stbl3* bacteria were subsequently transformed with the Golden Gate mixes in 96-deep-well blocks and grown for ∼20 h at 37°C (Fig. 2B and Supplementary Fig. 1C). In order to equalize bacterial growth among wells and obtain consistent plasmid yield, cultures were diluted 1:5 in fresh medium and grown for additional 3.5 h. Analysis of bacterial growth by spectrophotometric measurements indicated uniform cell growth across wells (Supplementary Fig. 1D,E). Analysis of selected plates by Sanger sequencing of isolated plasmids confirmed the high efficiency and accuracy (99%) of the cloning procedure (Fig. 2C). Production of the entire library was completed in 10 days.

In addition to generating individual sgRNAs in an arrayed format, we also produced two types of pooled libraries, one containing all sgRNAs, to be used in selection screens (pooled library), and one in which all sgRNAs targeting the same gene were combined and maintained in a 96-well format (gene pools, in 450 wells), to be used in high-content screens (Fig. 2D). Both pooled libraries were generated by combining aliquots of bacterial culture after the additional 3.5 h of growth. All individual and pooled bacterial stocks were digitally tagged with barcodes and stored at −80°C (Fig. 2D).

### Assessment of sgRNA representation in the library

In order to assess the relative representation of sgRNAs in the generated library, we performed high-throughput sequencing of the pooled library and compared relative abundance of each guide. Eighty-seven percent of the designed sgRNAs were detected, showing a narrow distribution of read counts (Fig. 2E). The raw aligned reads for the majority of sgRNA sequences present in the pool showed less than 2-fold difference from the mean (Fig. 2E). A single outlier overrepresented compared to the other sgRNAs was found to have contaminated neighboring wells in one plate (Fig. 2E). Sanger sequencing of individual plasmids isolated from re-grown glycerol stocks allowed us to identify the contaminated wells and replace them with the correct plasmids.

Undetected sgRNAs were re-cultured from bacterial glycerol stocks in individual wells of 96-well blocks, and equalized to an OD600 of ∼0.80 prior to plasmid extraction. Sanger sequencing indicated that 58% of the sgRNAs undetected in the pooled library had been successfully cloned and had the correct sequence, suggesting that they may have only been underrepresented in the pooled library. Purified plasmid DNA from the originally underrepresented wells was added to the pooled library. The final library comprised 94% of the designed guides allowing targeting of all 450 target genes, with 99% of genes targeted by at least 4 sgRNAs (Fig. 2F, Supplementary Table 2).

### Assessment of sgRNA activity

**Figure 3.**
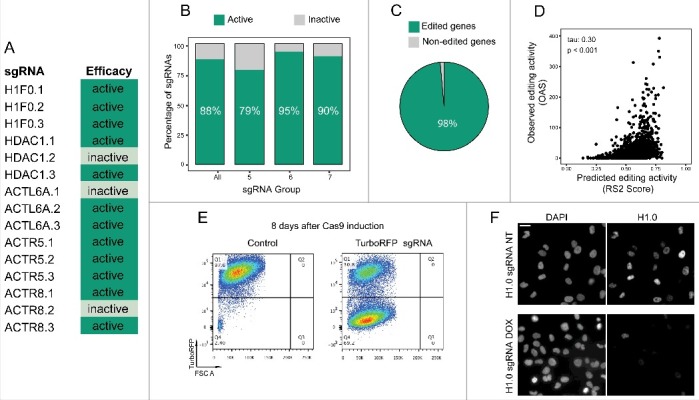
**Validation of the sgRNA library A.** Assessment of sgRNA in/del activity of the indicated guides. Assessment of activity was based on Sanger sequencing of PCR-amplified and cloned target regions. **B**. Assessment of sgRNA in/del efficacy of all guides included in the indicated sgRNA groups. Assessment of activity was based on next generation sequencing of pulled-down target regions. The indicated percentage of active sgRNA is calculated based on the number of sgRNAs detected in the infected cells. **C**. Percentage of genes edited by at least 1 of the 3 tested sgRNAs. **D**. Correlation between the observed and predicted sgRNA editing activity. Tau indicates the Kendall rank correlation coefficient (tau-b) **E**. FACS analysis of cells expressing TurboRFP in the presence (right) or absence (left) of TurboRFP-targeting sgRNAs. **F**. Immunofluorescence microscopy of uninduced (NT) or induced (DOX) cells expressing *H1F0*-targeting sgRNAs. Cells were analyzed 14 days after Cas9 induction. Scale bar: 10 µm.

To assess the editing efficacy of the generated library, we initially selected 15 sgRNAs and examined their ability to induce in/dels at their target sites. To do so, we transduced human fibroblasts expressing inducible Cas9 with individual constructs and assessed in/del formation by TOPO cloning of PCR–amplified genomic fragments of the targeted loci. Fourteen days after Cas9 induction, 12/15 targeted sites showed in/dels, indicating overall high editing activity (Fig. 3A). Time course experiments indicated that in/del formation increased over time after Cas9 induction, typically reaching a plateau at 8 days (not shown).

To comprehensively assess the ability of individual sgRNAs to induce in/del formation at all target genes, we generated 3 pools of 450 sgRNAs containing one sgRNA/gene each (pool 5, pool 6, and pool 7, each one containing distinct sgRNAs for the same gene) (Supplementary Table 2), and transduced Cas9-expressing HepG2 cells with the three individual pools. Five days after infection, genomic DNA was extracted and targeted sites pulled-down using target-enrichment probes (see methods). Detection of in/dels by high-throughput sequencing confirmed high editing activity for most sgRNAs. Eighty-seven percent of sgRNAs induced detectable in/dels and 98.6% of the genes were targeted by at least one sgRNA (Fig. 3B,C). sgRNA groups 6–7 showed higher activity (95% and 90% of active sgRNAs, respectively) compared to sgRNA group 5 (79%) (*P* value <0.0001 Fisher's test), suggesting that the algorithm used by Wang et al. to design sgRNAs may outperform the MIT CRISPR Designer with respect to editing efficacy (Fig. 3B). However, higher sgRNA activity would not necessarily result in higher KO efficiency since sgRNA groups 6–7 do not preferentially target 5’ exons, unlike sgRNA groups 1–5 (Fig. 1D), and may lead to long truncated proteins retaining partial functionality. Thus, the combination of multiple sgRNAs designed with different algorithms maximizes the chance of achieving efficient gene KO with our library.

Comparison between the 50 most active sgRNAs in each sgRNA group and those showing low or undetectable activity indicated that the number of in/dels induced by individual sgRNAs did not correlate with abundance of the sgRNA in the pool, GC content of the target sequence or specificity score (Supplementary Fig. 2C). To quantify the editing activity of each sgRNA present in the library, we calculated an observed activity score (OAS: the number of observed in/dels normalized to the abundance of each sgRNA in the library—see Materials and methods) (Supplementary Table 2). The OAS showed a significant, albeit weak, correlation with the Azimuth RS2 score (Kendall rank correlation coefficient = 0.3, *P* <0.001), indicating that the algorithm is overall predictive of sgRNA activity, although it returns many false positives and false negatives (Fig. 3D, Supplementary Fig. 2C). Similar results were obtained comparing the in/del score with the sgRNA Scorer 2.0 (Supplementary Fig. 2D). Thus, while *in silico* analysis can help identify active sgRNAs, actual editing efficacy needs to be empirically determined.

High sgRNA efficacy was confirmed at the protein level. To perform an initial quantitative assessment of KO kinetics and efficiency in polyclonal populations, we used a reporter cell line expressing TurboRFP. FACS analysis of reporter cells transduced with TurboRFP-targeting sgRNAs indicated rapid and efficient KO at the protein level, detecting ∼70% RFP-negative cells 8 days after Cas9 induction (Fig. 3E). KO of the endogenous gene *H1F0* using sgRNAs from the library showed similar results (90% of H1.0-negative cells, as assed by immunofluorescence microscopy) (Fig. 3F). Thus, stable expression of sgRNAs allows efficient gene KO in polyclonal populations, avoiding the need of isolating individual clonal populations of edited cells.

### Sensitive detection of phenotypic effects induced by gene knock-out in polyclonal populations

**Figure 4.**
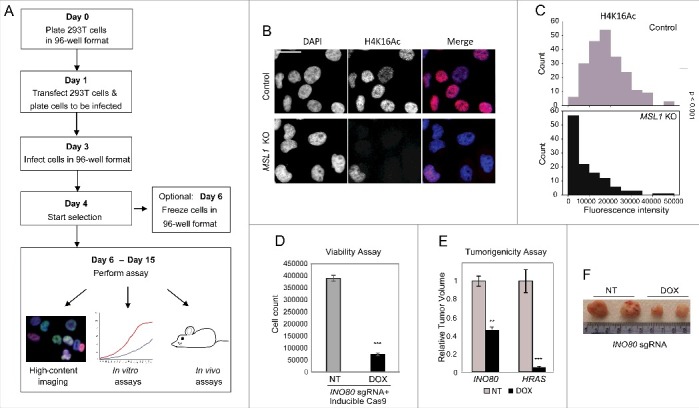
**Applications of the arrayed sgRNA library A**. Outline of the protocol for large-scale gene editing and subsequent functional assays. **B-C**. Quantitative immunofluorescence microscopy of cells in which KO of *MSL1* had been induced for 14d and control cells not expressing *MSL1* sgRNAs (control). *MSL1* is a member of the MSL complex, which specifically acetylates H4K16. For quantification, n = 218 and 123 for control and *MSL1* KO, respectively. *P* value: Mann–Whitney test. Scale bar: 10 µm**. D**. Viability assay comparing uninduced (NT) and induced (DOX) cells expressing *INO80*-targeting sgRNAs and inducible Cas9. n = 3. Three asterisks indicate *P* < 0.001 (Student t-test). **E-F**. Tumorigenicity assays in NSG mice comparing uninduced (NT) and induced (DOX) cells expressing *INO80*- or *HRAS*-targeting sgRNAs and inducible Cas9. n = 4. The final tumor volume relative to the corresponding uninduced condition is indicated in the graph. Tumors were harvested 11 weeks after injection. Two and three asterisks indicate *P* < 0.05 and *P* < 0.001, respectively.

CRISPR-mediated KO offers major advantages compared to RNAi-based approaches, allowing complete elimination of the targeted protein. However, the low editing efficiency achieved when using transiently expressed sgRNAs and the consequent need to isolate clones limits the use of CRISPR for high-throughput approaches. Furthermore, the inability to temporally control induction of gene KO when Cas9/sgRNA complexes are transiently expressed, hinders some applications, especially if essential genes are targeted (e.g., *in vivo* studies). Using the platform that we have generated, gene KO can be induced in polyclonal populations in a time-controlled manner, allowing large scale generation of cell lines suitable for a variety of *in vitro* and *in vivo* assays (Fig. 4A). To confirm that the functional consequences of gene KO are robustly detected in polyclonal cell populations, we first assessed whether interference with a histone modifying protein complex led to changes in the corresponding histone mark. To do so, we disrupted the MSL complex, which specifically acetylates histone H4 lysine K16 [32], by knocking-out *MSL1*. To maximize the KO efficiency we transduced cells with the pool of *MSL1*-targeting sgRNAs that we generated. Upon *MSL1* KO, we observed a significant reduction in the levels of H4K16Ac (*P* value<0.001 Student t-test), confirming MSL1 loss-of-function and indicating that the molecular consequences induced by *MSL1* KO were readily detectable (Fig. 4B,C).

We then asked whether we could observe physiological consequences induced by gene KO using polyclonal populations. The gene encoding the chromatin remodeler *INO80* is an essential gene, whose loss leads to embryonic lethality and impairs cell proliferation [33,34]. In agreement, *INO80* KO inhibited cell growth and 8 days after Cas9 induction we observed a 7-fold reduction in the number of viable cells compared with the uninduced control (Fig. 4D).

Finally, to assess whether gene KO induced with our platform is a suitable approach to perform *in vivo* functional studies, we tested the effect of *INO80* KO in xenograft assays. To do so, *HRAS*^v12^-transformed fibroblasts [35] expressing inducible Cas9 and *INO80*-targeting sgRNAs, were injected intradermally into immunocompromised mice. When tumors became palpable (∼3 weeks after injection), doxycycline (Dox) was administered to mice to induce Cas9 expression and gene KO *in vivo*. In agreement with previous reports [34], loss of INO80 significantly inhibited tumor growth (Fig. 4E,F). As a positive control, *HRAS* KO almost completely prevented tumor growth, indicating that cells escaping gene KO, which are positively selected during tumor growth, are only a small fraction of the injected cells and do not significantly reduce the dynamic range of detectable differences (Fig. 4E).

## Discussion

We have generated and characterized a focused sgRNA library targeting most human epigenetic regulators that enables comprehensive investigation of cellular mechanisms involving chromatin and DNA methylation.  We show overall high editing activity of the sgRNAs contained in the library and provide a score of actual activity for 3 guides/gene to ease selection of the most effective sgRNAs. The library can be used for a variety of applications, ranging from loss-of-function screens to investigation of selected proteins *in vitro* and *in vivo*, visualization of genomic loci [13] and induction of genomic rearrangements at specific loci [10].

The library is available in three formats: a pool containing all sgRNAs, suitable for both enrichment and drop-out selection screens; an arrayed format in which multiple sgRNAs targeting the same gene are combined, suitable for primary high-content screens; an arrayed format containing individual sgRNAs, which can be used to validate primary hits or to investigate selected proteins in a targeted manner. Importantly, we show that the high editing efficiency in polyclonal populations allows assessment of functional consequences of gene KO without the need of isolating clones. This removes a time-consuming step and generates more reliable data, avoiding clonal effects that can significantly affect the biological interpretation of the results, especially when heterogeneous cell populations such as cancer cell lines are used.

Compared with alternative resources, our library offers various advantages. First, the focused nature of the library enables effective screening, even when complex readouts, such as clonogenic or invasion assays, or microscopy-based measurements are used. While a pooled genome-wide library requires ∼200 million cells to cover its complexity 1000 times [18], only 3 million cells are enough to saturate the screen with our focused library. Similarly, for arrayed screens, the use of our library reduces the number of required plates from 383 96-well plates [24] to 5 when using the gene pools, or to 33 when using the individual sgRNAs. Second, the presence of 7 sgRNAs for most targeted genes increases the confidence in the results and minimizes the rate of false-positives compared with genome-wide arrayed libraries containing only 2 sgRNAs per gene [24], while the design of guides with distinct strengths (targeting 5’ of the gene or optimized activity) maximizes overall efficacy. Third, being cloned into a lentiviral vector that can be easily propagated, the generated library is a cost-effective alternative to synthetic arrayed libraries [36]. Fourth, the ability to temporally control gene KO when combined with inducible Cas9 enables the use of sgRNAs *in vivo*, an important benefit when using graft models.

In conclusion, the generated CRISPR library represents a unique resource to study epigenetic mechanisms in various contexts, allowing both forward and reverse genetic loss-of-function screens and targeted investigation of selected proteins *in vitro* and *in vivo*.

## Materials and methods

### Cell lines

*In vitro*-transformed fibroblasts [35] were cultured in minimal essential media (MEM) with 15% fetal bovine serum (FBS), HepG2 cells were cultured in MEM with 10% FBS, and HEK-293T cells were cultured in Dulbecco's Modified Eagle's Medium (DMEM) with 10% FBS. Media was supplemented with 2 mM L-glutamine, 100 U/ml penicillin, and 100 μg/ml streptomycin. To freeze cells in 96-well format, cells were detached from the plate using 30 μl of trypsin per well. Following addition of 70 μl of FBS containing 10% DMSO, plates were sealed and stored at -80°C for up to 4 weeks. To thaw cells, plates were placed in a water bath at 37°C for a few seconds and spun for 5 min at 4°C after addition of 50 μl of medium to each well. Fresh medium (100 μl) was finally added to each well after removal of 120 μl of freezing medium.

For generation cell lines expressing 3xFLAG-tagged inducible Cas9, transformed fibroblasts and HepG2 cells were transduced with a lentiviral pCW-Cas9 vector [3,14]. (Addgene), which was modified to confer resistance to hygromycin to infected cells. Following a 7-day selection with 600 μg/mL hygromycin, cells were plated at a very low density (200 cells/15 cm plate) and grown for 2–3 weeks. Clones with minimal background levels of Cas9 and responsive to induction with 1 μg/mL doxycycline were identified by both RT-qPCR of Cas9 expression and western blotting of 3xFLAG-tag (Sigma F1804). To generate cell lines stably expressing sgRNAs, cells were transduced with pLenti_BSD_sgRNA constructs and selected with blasticidin (4 μg/ml) for 5 days. To induce gene KO, Cas9 expression was induced by the addition of doxycycline (1 μg/ml).

### Library design

#### sgRNA selection

Where possible, sgRNAs were designed to target the first shared CDS exon of identified genes (RefSeq). The most 5’ exon of RefSeq variant 1 was utilized if it was not possible to target a shared exon given a specific scoring threshold. For later alignments, the RefSeq variant 1 was used as a reference. Genomic CDS sequences—separated into individual exons—associated with RefSeq accessions for all gene targets were extracted using the biomaRt package for *R* [37]. CDS sequences were extracted manually from UCSC genome browser in 13 cases where the biomaRt database could not identify the input RefSeq accession. GRCh37/hg19 (February 2009 assembly) was used as the reference genome throughout the design stage. Five sgRNAs per gene (Henser sgRNA groups 1–5) were evaluated for off-target activity using the Zhang lab's publicly available tool at crispr.mit.edu (accessed between April and October 2015). Guides were selected using a score threshold of at least 70 where possible, with guides scoring at least 50 used as backups. For each gene in the library, 2 additional guides were selected from the CRISPR library constructed by Wang et al. (Wang sgRNA groups 6–7) [18], assuring that selected sequences did not overlap with existing guides by at least 15 bp. The sgRNA design by Wang et al. is based on observed activity of sgRNAs targeting essential ribosomal genes in depletion screens. Briefly, sgRNAs with high predicted activity were designed using a support-vector-machine classifier that identified up to 80 binary features of efficient guides [18]. Overall, our resulting library contained 3150 unique sgRNA sequences with 7 sgRNAs per target gene. A 5′ ‘G’ nucleotide is commonly added to sgRNAs expressed under a U6 promoter for efficient RNA polymerase III transcription; this additional step was not required in this case as a 5′ ‘G’ nucleotide is prepended to all protospacer sequences automatically in the vector during cloning.

#### Efficacy prediction

On-target efficacy of sgRNAs was predicted *in silico* using both Rules Set 2 (RS2) from the Azimuth project [29], and sgRNA Scorer 2.0 (SS2) [30] prediction models using available python scripts [RS2 v1.2 (accessed August 2016), SS2 v2 (accessed May 2017)]. In the case of RS2 only the raw sequence option (-*-seq*) was used in score calculations in the format of: NNNN20merNGGNNN. The percentage of peptide remaining following a DSB was predicted by mapping sgRNA sequences against canonical mRNA sequences, assuming a frame-shift or nonsense mutation would be induced at the final nucleotide before the PAM sequence.

### Large-scale sgRNA cloning

#### Lentiviral vector

The lentiviral vector used to construct this sgRNA library (pLenti_BSD_sgRNA) was generated through modification of the pLent_sgRNA_Lib plasmid utilized in previously published CRISPR/Cas9 screens [31], which we obtained through Addgene. The original GFP reporter was replaced with the blasticidin resistance coding region (BSD) using GeneArt Seamless Cloning and Assembly.

#### Library oligo synthesis and annealing

Forward and reverse oligonucleotides corresponding to the target sequences were tagged with 5′ overhangs appropriate for the *BsmBI*-based cloning strategy. Single-stranded complementary forward and reverse oligonucleotides were synthesized at 100μM in a 96-well arrayed format by Sigma Aldrich—resulting in 66 (2 × 33) 96-well plates. To generate double-stranded oligonucleotides, relevant pairs were mixed and added to annealing buffer (10 mM Tris pH 7.5, 1 mM EDTA, 50 mM NaCl) using the Biomek FX automated liquid handling equipment, maintaining the 96-well arrayed format (Supplementary Table 3). Plates of mixed oligos (x33) were then annealed in thermocyclers by heating the reaction to 95°C for 5mins before reducing the temperature to 25°C at 0.1°C per second. Following the reaction, annealed oligonucleotides were diluted 1:200 before proceeding to Golden Gate cloning.

#### Cloning and transformation

Cloning of double-stranded oligonucleotides into pLenti-BSD-sgRNA was performed using a single-step Golden Gate cloning procedure (Supplementary Fig. 1B). Annealed and diluted oligonucleotides from the previous step were added to each well of a fresh 96-well PCR plate maintaining the original arrayed format and combined with the cloning mix (Supplementary Table 3). Reaction PCR plates were placed in thermocyclers and the cloning reaction performed over the course of ∼2 h using the conditions indicated in Supplementary Fig. 1. All plates were stored at -20°C until transformation.

Chemically competent *E. coli Stbl3* with a transformation efficiency of 1.5 × 10^7^ cfu/μg DNA were generated using the Mix & Go *E. coli* Transformation Kit (Zymo Research). To transform the sgRNA library, competent bacteria were thawed and 50 μl added to each well of 96-well deep well blocks. Subsequently, 2 μl of constructs generated in 96-well PCR plates during the cloning step were transformed directly into these wells. All transformation steps were conducted on ice. CircleGrow medium (950μl; MP Biomedicals) was added to each well, and blocks were initially incubated overnight (ON) for ∼20 h at 37°C. Individual glycerol stocks were generated (33 96-well plates). All individual glycerol stocks were digitally tagged with Data Matrix 2D barcodes for simplified tracing and stored at -80°C.

#### Generation of library pools

To generate the whole library pool, 200 μl of culture from wells grown ON were transferred into the corresponding wells of new deep-well blocks, and 800 μl of fresh medium added to each. The new deep-well blocks were cultured for a further 3.5 h to equalize their optical density (OD) and respective plasmid yields (Supplementary Fig. 1D). A total of 200 μl of the equalized culture from each well were then pooled generating ∼700 ml of culture used to extract plasmid DNA. To generate gene pools, 50 μl of equalized culture from each well corresponding to the same gene were pooled together and used to generate glycerol stocks (5 plates, 450 wells, with each well containing all sgRNAs targeting the same gene).

### Viral production and multiplicity of infection (MOI) estimation

To optimize conditions for viral transduction, virus was produced in parallel in 10 cm dish- and 96-well format. For 10 cm dish-format, lentiviral particles were produced by transfecting HEK293T packaging cells at 80% confluence with 5 μg of the sgRNA lentiviral construct, 3.75 μg of psPax2 packaging plasmid, 1.25 μg of pMD2G envelope plasmid and 1 μg pAdVantage (Promega E1711) at a ratio of 3:1 DNA to FugeneHD (Promega E2311). For 96-well format, the number of cells and the amount of plasmid DNA was reduced 40 times. Approximately 24 h and 48 h after transfection, the supernatant containing viral particles was recovered, filtered through a 0.45 μm filter (Millipore SLHV033RS or MSHVS4510), pooled together, and frozen at -80^o^C. In order to determine the multiplicity of infection (MOI) of the virus, 1 × 10^5^ fibroblasts were seeded per well of a 6-well plate and infected with serial dilutions of virus generated in 10 cm dish-format, starting from 1:1. Following selection with 4 μg/mL blasticidin, the number of viable cells/well were measured and compared to a non-infected control to calculate the percentage of infected cells. The formula P = 1-e^−m^, where P is percentage of infected cells and m represents MOI, was used to determine the viral concentration yielding the desired MOI in 96-well format (typically MOI = 1).

### Individual sgRNA validation

Initial assessment of sgRNA activity was performed by transducing individual sgRNAs into fibroblasts containing a doxycycline-inducible Cas9. Cells (2 × 10^6^) were plated in 10 cm dishes and infected with pLenti_BSD_sgRNA lentivirus for 24 h. Transduced cells were selected with blasticidin for 4 days, after which Cas9 was induced for 1–2 weeks. KO was assessed via FACS, quantitative immunofluorescence microscopy, and/or TOPO-cloning and Sanger sequencing of sgRNA target genes. For TOPO sequencing, primers were designed to amplify genomic DNA sequences surrounding predicted sgRNA target sites (Supplementary Table 4). PCR was performed on genomic DNA from cells expressing Cas9 and respective sgRNAs, and amplicons cloned into blunt-end TOPO vectors. Resultant TOPO-vectors were transformed into *Stbl3* competent *E. coli*, and DNA extracted for sequencing. Standard M13 sequencing primers were used to sequence the cloned amplicons. For FACS analysis a cell line transduced with empty pTRIPZ (Open Biosystems) and expressing TurboRFP was used. FACS analysis and immunofluorescence analysis was performed as previously described [38]. Antibodies used were anti-H1.0 (Upstate) and anti-H4K16Ac (Cell Signaling Technology). Quantification of fluorescence intensity was performed using Metamorph software.

### Large-scale sgRNA validation

To assess the efficacy on a larger scale, individual sgRNAs separate pools were generated for sgRNA groups 5, 6, and 7, resulting in 3 pools, each containing a single sgRNA for each of the 450 target genes. HepG2 cells containing a doxycycline inducible Cas9 were plated at 0.45 × 10^6^ cells/well in a 6-well plate and were induced for 24 hours prior to infection with the above pools in duplicate. Cells were infected with virus at MOI ∼ 10. 24 hours after infection, cells were transferred into 60mm dishes and selected with blasticidin for 4 days. Doxycycline was replaced every 2 days until harvesting of genomic DNA for sequencing 5 days post-infection.

### Next generation sequencing

To assess the representation of individual sgRNAs in the whole library and in the pools, libraries of amplicons containing the sgRNA sequences were generated by PCR using the Herculase II polymerase kit (Agilent). Briefly, 150 ng of the pooled plasmid libraries were used to generate 268 bp-long amplicons, which included the sgRNA sequence and the P5 and P7 adaptors for Illumina sequencing. All primers were annealed at a temperature of 60°C for 30 seconds and the reaction performed for 30 cycles. All reactions were performed in technical triplicates and their products pooled and run on a 2% agarose gel to confirm a single band of 268 bp. The bands were subsequently cut from the gels and purified using the gel purification Kit (Qiagen) as per manufacturer's protocol. Purified products were sequenced with a HiSeq2500 using custom sequencing and indexing primers (SeqP and IndexP, Supplementary Table 4). Following sample demultiplexing, all sgRNA sequences were trimmed and aligned to the target sequences to assess sgRNA representation (normalized read count). To detect in/dels at targeted regions, libraries were generated using the SureSelect Target enrichment kit (Agilent) using custom probes and following the manufacturer's instructions. Probes were designed to cover 2 kb regions centered on each target site. When multiple target sites were located in the same exon, the 2 kb region was centered on the exon middle point. Probe tiling parameters were: Tiling density: 1x; Masking: Least Stringent; Boosting: Maximize Performance.

### Large-scale in/del analysis

The quality of the sequenced reads was assured using FastQC (https://www.bioinformatics.babraham.ac.uk/projects/fastqc/). Alignment was carried out against the UCSC hg19/GRCh37 genome assembly, using BBMap (v. 36.59) (http://jgi.doe.gov/data-and-tools/bbtools/bb-tools-user-guide/bbmap-guide/), a global aligner that is able to align longer in/dels. A two-stage alignment strategy was used to robustly identify reads that contained in/dels. In the first phase, reads were aligned to the genome disallowing any reads that contained in/dels. In this phase, reads that aligned in a proper pair (unedited or correctly repaired sequences) were discarded and the remainder was taken forward. In the second phase, the remaining reads were aligned to the genome, allowing in/dels up to 2000 bp. Duplicates were marked using Picard (v. 2.1.0). Reads that were marked as duplicates, or that had a mapping quality score of less than 38 were filtered using samtools (v. 1.2) [39] and sambamba (v. 0.6.0) [40]. This two-phase approach was necessary to discriminate between background noise and signal in our dataset since infection with pooled sgRNAs returned a high proportion of unedited sequences for each target sequence (only a small fraction of cells was transduced with each sgRNA). All subsequent downstream analysis of the reads was performed in R (v. 3.3.2). The location and size of in/dels in reads were identified from the CIGAR string. In/dels were only considered valid if they occurred within 2 nucleotides of the Cas9 cleavage site (defined as 6 nucleotides upstream of the end of the guide RNA including the PAM sequence). Any in/dels that could also be detected in the untransduced HepG2 sample were removed from the analysis. To calculate the ‘*observed activity score’* (OAS), the number of in/dels observed for each sgRNA was normalized to its representation in the pools: the mean number of observed in/dels across two biological replicates (μID) was divided by the normalized read count for that sgRNA [raw reads for a single sgRNA (sgR) divided by total reads of all sgRNAs in the pool (TR) * 100].

$$OAS=\frac{\mu ID}{(sgR÷TR)*100}$$

### Viability and tumorigenicity assays

To assess the functional consequences of CRISPR-mediated KO induced by sgRNAs in our library, transformed human fibroblasts expressing inducible Cas9 were transduced with sgRNAs against either *INO80* (a pool of 7 sgRNAs) or *HRAS* (1 sgRNA). Following selection of infected cells with blasticidin, the resulting polyclonal populations were used for phenotypic validation. For viability assays, cells transduced with *INO80*-targeting sgRNAs were seeded in triplicates at 100,000 cells/well in a 6-well plate and cultured in the presence or absence of doxycycline (1 μg/mL) for 8 days. The final number of cells/well was determined by manual cell counting in the presence of Trypan Blue to discard dead cells. For functional *in vivo* assays, 5 × 10^5^ transformed fibroblasts transduced with *INO80*-targeting sgRNAs and expressing inducible Cas9 were injected intradermally together with 1 × 10^5^ carrier hTERT-immortalized fibroblasts in 50 µl of PBS into both flanks of 6–8 week old male NOD/SCID/Interleukin 2 receptor γ null (NSG) mice. After 3 weeks, when tumors became palpable, mice were randomly grouped and administered either 2 mg/mL dox in 1% sucrose water or 1% sucrose alone. Six weeks after treatment, tumor volume was measured according to the formula d^2^*D/2, where d and D are the shortest and the longest diameter of each tumor, respectively. Cells expressing *HRAS*-targeting sgRNAs were used as a control. Animal studies were conducted in accordance with the Crick project license PPL 70/8167 approved by the Home Office.

### Statistical analyses

All data visualization and statistical analyses were conducted using the *ggplot2* and *stats* packages for *R*, or MS Excel. The test used for each analysis is indicated in the figure legend.

## Data availability

The datasets generated during and/or analyzed during the current study are available from the corresponding author on reasonable request

## Supplementary Material

Sup-mat-Generation_of_an_arrayed_CRISPR-Cas9_-Henser-Brownhill.rar
